# Blocking IL-25 signalling protects against gut inflammation in a type-2 model of colitis by suppressing nuocyte and NKT derived IL-13

**DOI:** 10.1007/s00535-012-0591-2

**Published:** 2012-04-27

**Authors:** Ana Camelo, Jillian L. Barlow, Lesley F. Drynan, Daniel R. Neill, Sarah J. Ballantyne, See Heng Wong, Richard Pannell, Wei Gao, Keely Wrigley, Justin Sprenkle, Andrew N. J. McKenzie

**Affiliations:** 1 MRC Laboratory of Molecular Biology, Hills Road, Cambridge, UK; 2 Present Address: Department of Clinical Infection, Microbiology and Immunology, Institute of Infection and Global Health, University of Liverpool, Liverpool, L69 7BE UK; 3 Present Address: MedImmune, Milstein Building, Granta Park, Cambridge, CB1 6GH UK; 4 Centocor Research and Development, a division of Johnson & Johnson Pharmaceutical Research & Development, L.L.C., 145 King of Prussia Road, Radnor, PA 19087 USA

**Keywords:** IL-25, Inflammatory bowel disease, Nuocytes, IL-13

## Abstract

**Background:**

Interleukin-25 (IL-25) is a potent activator of type-2 immune responses. Mucosal inflammation in ulcerative colitis is driven by type-2 cytokines. We have previously shown that a neutralizing anti-IL-25 antibody abrogated airways hyperreactivity in an experimental model of lung allergy. Therefore, we asked whether blocking IL-25 via neutralizing antibodies against the ligand or its receptor IL-17BR could protect against inflammation in an oxazolone-induced mouse model of colitis.

**Methods:**

Neutralizing antibodies to IL-25 or IL-17BR were administered to mice with oxazolone-induced colitis, a model of ulcerative colitis. The disease onset was evaluated by weight loss and degree of colon ulceration. Also, lamina propria and mesenteric lymph node (MLN) infiltrates were assessed for mucosal inflammation and cultured in vitro to determine cytokine production.

**Results:**

We found that in oxazolone colitis IL-25 production derives from intestinal epithelial cells and that IL-17BR^+^ IL-13-producing natural killer T (NKT) cells and nuocytes drive the intestinal inflammation. Blocking IL-25 signalling considerably improved the clinical aspects of the disease, including weight loss and colon ulceration, and resulted in fewer nuocytes and NKT cells infiltrating the mucosa. The improved pathology correlated with a decrease in IL-13 production by lamina propria cells, a decrease in the production of other type-2 cytokines by MLN cells, and a decrease in blood eosinophilia and IgE.

**Conclusion:**

IL-25 plays a pro-inflammatory role in the oxazolone colitis model, and neutralizing antibodies to IL-25 or IL-17BR can slow the ongoing inflammation in this disease. Because this model mimics aspects of human ulcerative colitis, these antibodies may represent potential therapeutics for reducing gut inflammation in patients.

## Introduction

Inflammatory bowel diseases (IBDs), such as Crohn’s disease (CD) and ulcerative colitis (UC), are inflammatory disorders of the digestive tract that occur due to a dysfunctional immune response to usually harmless commensal bacteria [1]. These immune responses have been broadly divided into Th1, Th2, or Th17-type responses depending on the T-helper cell type driving the response and the respective cytokine profile. Generally, CD is characterized by a transmural, discontinuous inflammation that is associated with a type-1 response mainly driven by interleukin (IL)-12 and interferon (IFN)-γ [2, 3], while UC involves the superficial mucosal and submucosal layers of the colon and is driven by type-2 cytokines, such as IL-4, IL-5, and IL-13 [1, 4]. IL-4 and IL-5 expressions were previously quantified in intestinal tissue and correlated with the clinical and histological severity of colitis in UC patients [5], and IL-13, which was also found to be up-regulated in UC, was linked to an impaired epithelial barrier function in the gut [6]. In mice, mucosal inflammation can be induced by the administration of dextran sulfate sodium (DSS) or haptenating agents, such as 2,4,6-trinitrobenzenesulfonic acid (TNBS) and oxazolone [7]. These agents result in very different types of inflammation in the gut, with the first two skewing towards a type-1 phenotype [8, 9], whereas the oxazolone model shows a clear type-2 inflammatory response [10–12].

IL-25 was originally found by sequence homology to other IL-17 family members [13], although unlike the other IL-17 cytokines, IL-25 function has been associated with type-2-like inflammation [14, 15]. Expression of IL-25 has been reported in several tissues, including lung, stomach, small intestine, and colon [14] and in cell types such as macrophages and epithelial cells in the gut [14, 16], Th2 cells [14], mast cells [17], and natural killer T (NKT) cells [18]. IL-25 mediates its biological effects through the receptor IL-17BR, which forms a receptor complex with IL-17RA, both being essential for IL-25 effector functions in the intestine [19]. IL-25-induced inflammation is typically characterized by elevated levels of type-2 cytokines which lead to pathological changes in the lungs and digestive tract, such as elevated serum IgE and IgG1, increased mucus secretion, and epithelial cell hyperplasia [20–22]. A key role for IL-25 in driving lung inflammation during allergic asthma has been widely reported [23–25], with its expression up-regulated in the nasal lavage fluids of asthmatic patients [26]. Moreover, in a mouse model of lung inflammation, we have shown that blocking IL-25 with a neutralizing anti-IL-25 antibody completely abrogated airways hyperreactivity (AHR) [23]. In another mucosal context, the intestine, IL-25 has been described to play a role in the protection against helminth infection [27] by inducing a strong type-2 innate inflammation which is dependent upon the activation of IL-17BR^+^ nuocytes and their production of IL-13 [28]. In gut inflammation, such as colitis, an anti-inflammatory role has been attributed to IL-25 in type-1 models of this disease by two separate groups [16, 29], but an exact mechanism of how IL-25 acts to prevent gut inflammation in these models has not been described in full, although IL-25 was associated with the regulation of IL-12 production and induction of alternatively activated macrophages with anti-inflammatory properties [30]. In another model, severe intestinal inflammation following chronic *Trichuris* *muris* infection was shown to develop in mice that were IL-25-deficient, correlating with a heightened expression of Th1/Th17 cytokines, IFN-γ, and IL-17 [31]. Despite these findings, there is so far no record of an anti-inflammatory role for IL-25 in type-2-skewed colitic inflammation. Type-2 gut inflammation is not driven by IL-12 and IFN-γ, but is thought to be caused by elevated levels of IL-4, IL-5, and IL-13 [32] and studies have shown a delay in the onset of colitis when IL-4 is blocked by a neutralizing anti-IL-4 antibody [11]. Similarly, when IL-13 downstream effector functions are blocked, via the elimination of NKT cells or by using an IL-13 receptor α2-fusion protein, the development of colitis is prevented [10], whereas antibodies against IL-12 severely aggravate the disease [11]. In two recent studies, another type-2-associated cytokine, IL-33, has been associated with UC [33, 34], pointing to different inflammatory outcomes depending on which type of IBD is being studied. Therefore, because IL-25 is known for driving IL-13 production by IL17BR^+^ cells [28, 35] and for inducing type-2 inflammation in the lung and gut, we hypothesized that IL-25 might have a pro-inflammatory role in the type-2 model of colitis. We sought to investigate the role of IL-25 in type-2 gut inflammation using neutralizing antibodies against the cytokine and the IL-17B receptor. We demonstrate that IL-25 plays a critical role in mediating intestinal type-2 inflammation in oxazolone colitis and that, in contrast to the DSS and TNBS type-1 models, it acts as a pro-inflammatory cytokine. Our data suggest that IL-25 is essential for type-2 cytokine production by mesenteric lymph node (MLN) cells, as well as being essential for IL-13 production by leukocytes in the intestinal mucosa; this result supports previous findings in helminth infection and in lung inflammation [23, 27]. In addition, we also found that blocking IL-25 signalling decreased the number of inflammatory cell types such as nuocytes and NKT cells in the lamina propria.

## Materials and methods

### Animals

Wild-type female BALB/c mice were obtained from Charles River Laboratories (Margate, UK). All mice were maintained in a specific pathogen-free environment. Experiments were conducted under license from the United Kingdom Home Office.

### Induction of colitis and antibody treatment

Oxazolone (4-ethoxymethylene-2-phenyl-2-oxazoline-5-one) was obtained from Sigma-Aldrich (St. Louis, MO, USA). Induced gut inflammation was performed according to a recently described method of sensitization and challenge [10]. To pre-sensitize mice, a 2 × 2 cm field of the abdominal skin was shaved and 150 μl of a 4 % (w/v) solution of oxazolone (OXA) in 100 % ethanol (EtOH) was applied. Seven days after pre-sensitization mice were challenged intrarectally with 150 μl of 3 % OXA in 50 % EtOH, under anesthesia with isoflurane. Control mice received EtOH only. Neutralization of IL-25 signalling in vivo was performed at 500 μg/mouse with monoclonal antibodies (mAbs) against IL-25 (Clone 2c3.1 or DDG91) [23] or IL17BR (Clone D9.2), made in house [28]. The isotype control used in this study was anti-c-myc mouse IgG1 (Clone 9e10.2). All antibodies were tested for endotoxin presence and used at concentrations lower than 0.01 EU/mg of antibody. Clinical symptoms were scored blind on day 1 and day 2 after intrarectal challenge and assigned clinical scores according to Table 1.

**Table 1:** Scoring system for disease activity index

Score	Weight loss (%)	Stool consistency/blood	Appearance/behavior
0	0	Normal	Normal/active
1	1–5	Slightly loose	Lack of grooming
2	5–10	Loose stools	Piloerection/subdued
3	10–15	Loose stools/blood	Splayed legs
4	15–20	Diarrhea/blood	Hunched/isolated

### Histological assessment of colitis

Colons were collected on day 2 post-intrarectal instillation and fixed in formalin (10 % formalin in 0.9 % saline solution). After paraffin embedding, 5-μm sections were cut and stained with hematoxylin and eosin (AML Labs, Baltimore, MD, USA). Stained sections were examined blind for evidence of colitis.

### Cell isolation and culture

MLN cells were isolated on day 2 after colitis induction by dispersing the tissue and filtering the cell suspension through a 70-μm cell strainer. Cells were cultured in RPMI 1640 supplemented with 10 % fetal bovine serum (FBS), 1 % penicillin and streptomycin, and 0.1 mM 2-mercaptoethanol. Lamina propria (LP) cells were isolated on day 2 from colonic tissue as previously described [36]. In brief, colon epithelial cells were isolated by the incubation of colon strips in RPMI/5 mM ethylediamine tetraacetic acid (EDTA). Epithelial cells were used for RNA extraction. Lamina propria (LP) cells were released by digestion of the tissue with RPMI/4-(2-hydroxyethyl)-1-piperazineethanesulfonic acid (HEPES) supplemented with 60 μg/ml DNaseI (Sigma), and 400 ng/ml of Liberase (Roche Applied Science, Burgess Hill, UK). Total LP cells were collected at this point for flow cytometric analysis. To isolate lymphocytes, cells were then collected by centrifugation in a discontinuous (40–100 %) Percoll gradient (GE Healthcare, Little Chalfont, UK). Finally, the lymphocyte-enriched population was further purified by negative selection using a CD45^+^-cell isolation kit (Miltenyi Biotec, Surrey, UK). For the cell cultures, lymphocytes were stimulated in vitro for 48 h with plate-bound anti-CD3 (1 μg/ml; BD Biosciences, Oxford, UK) and soluble anti-CD28 (1 μg/ml; BD Biosciences).

Isolation of nuocytes was performed by magnetic depletion of MLN cells and/or splenocytes using biotinylated antibodies against CD3 (145-2C11), CD19 (eBio1D3), FcεRI (MAR-1), and CD11b (M1/70). Negative selection was performed using magnetic cell sorting with streptavidin-conjugated beads (Dynabeads M-280; Invitrogen Dynal, Life Technologies Ltd, Paisley, UK). Cells were then incubated with IL-25 (R&D systems, Abington, UK) and anti-IL-25 or anti-IL17BR antibodies, at increasing concentrations. The cultures were kept for 3 days and the cell supernatants were used to measure IL-13 production by enzyme-linked immunosorbent assay (ELISA).

### ELISA analysis

Flat-bottomed 96-well ELISAmicrotiter plates (MAXISORP; Nunc, Roskilde, Denmark) were coated with 2 μg/ml of capture antibodies and held overnight at 4 °C. The plates were washed and blocked for 2 h with phosphate-buffered saline (PBS)/10 % FBS. For detection, 2 μg/ml of biotinylated detection antibodies were added for 2 h at room temperature before washing and incubating were performed with 1 μg/ml of streptavidin-horseradish peroxidase (SA-HRP), (MP Biomedicals, Solon, OH, USA). A peroxidase substrate, 2,2′-azinobis(3-ethylbenzthiazoline-6-sulfonic acid) (Sigma) was added following the manufacturer’s instructions. The signal was read spectophotometrically at 405 nm. All antibodies were purchased from BD Bioscience. These antibodies included affinity-purified anti-IgE (R35-72), anti-IgG2a (R19-15), anti-IL-2, anti-IL-4 (11B11), anti-IL-5, anti-IL-10 and anti-IFN-γ (XMG1.2). Biotinylated antibodies included anti-IgE (R35-72), anti-IgG2a (R19-15), anti-IL-2, anti-IL-4, anti-IL-5, anti-Il-10 and anti-IFN-γ. Standard curves were generated using recombinant mouse cytokines at 1:2 serial dilutions (R&D Systems). The IL-13 ELISA was performed using the Quantikine mouse IL-13 Immunoassay from R&D Systems. To test the specificity of the IL-17BR (clone D9.2) antibody, plates were coated with 1 μg/ml of IL-17BR-Fc, IL-17RA, IL-17RC-Fc, IL-17RD (SEF), or a control protein IL-13Rα-Fc. All proteins were purchased from R&D Systems. After blocking, anti-IL-17BR (clone D9.2) was used at varying concentrations, to test for binding. Detection was performed using biotinylated anti-mouse IgG1, SA-HRP, and a peroxidase substrate. The signal was read at 405 nm.

### Flow cytometry

All staining procedures were performed in PBS/10 % FBS at 4 °C. To block non-specific binding, the single-cell suspensions were incubated with anti-CD16/32 antibodies for 20 min. After washing, the cells were incubated with fluorochrome-conjugated antibodies for 30 min. Stained cells were analyzed on a FACS Calibur (BD Bioscience). Data analysis was performed using FlowJo software version 8.8.8 (Tree Star, Ashland, OR, USA). All antibodies used in flow cytometry were purchased from eBioscience (Hatfield, UK) and included anti-CD16/32 (clone 93), fluorescein isothiocyanate (FITC)-conjugated anti-Gr-1 (clone RB6-8C5), anti-CD4 phycoerythrin Cy7 (PECy7) (clone GK1.5), phycoerythrin (PE)-conjugated CD1d tetramer, anti-Siglec-F (clone E50-2440), and anti-γδ TCR allophycocyanin (APC) (clone eBioGL3). For the nuocyte staining we used a lineage combination, with PECy7-conjugated anti-CD3 (clone 145-2C11), CD19 (clone eBio1D3), CD11b (clone M1/70), Gr1 and B220 (clone RA3-6B2), and inducible T-cell costimulator (ICOS) conjugated to Alexa fluor 647(clone C398.4A). For the intracellular staining, cells were previously stimulated ex vivo with 50 ng/ml phorbol myristate acetate (PMA) and 500 ng/ml ionomycin for 4 h at 37 °C. All cells also received 1 μl/ml of Golgi plug (BD Bioscience). Following extracellular staining, cells were then fixed, permeabilized, and stained for IL-13 with anti-IL-13 PE- or APC-conjugated (eBio13A) according to the manufacturer’s instructions (BD Biosciences). For the IL-17BR surface staining, the in-house anti-IL-17BR antibody (clone D9.2) was conjugated with biotin and used in combination with streptavidin conjugated to either PECy7 or APCCy7.

### Differential white blood cell counts

Blood smears slides were stained with Giemsa (Sigma-Aldrich), following the manufacturer’s instructions. Differential cell counts were made blind on 100 cells per slide under oil at a 63× magnification.

### Quantitative real-time polymerase chain reaction (PCR)

RNA was prepared from lung tissues or from in vitro cultured primary cells using RNAzolB, according to the manufacturer’s instructions (AMS Biotechnology, Oxford, UK). Pre-designed Taqman™ primer and probe sets (Applied Biosystems, Carlsbad, California, USA) were used to quantify the expression of mouse *Il25* (Mm00499822_m1). Expression of *Il25* was quantified relative to that of *Gapdh* (mouse).

### Statistical analysis

Results are presented as means ± SEM. The statistical significance of differences between experimental groups was analyzed using two-way analysis of variance (ANOVA) for overall comparisons or the Student’s unpaired *t*-test for individual comparisons. *P* < 0.05 was considered as statistically significant. All analyses were performed using GRAPHPAD PRISM software (GraphPad Software, La Jolla, CA, USA).

## Results

### IL-25 expression is increased following oxazolone instillation

**Fig. 1:**
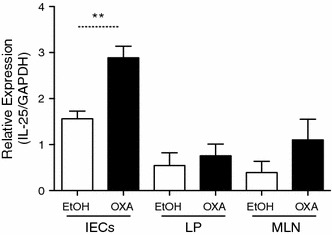
Interleukin-25 (*IL-25*) expression is upregulated during oxazolone colitis. Real-time polymerase chain reaction (PCR) for IL-25 RNA transcripts in intestinal epithelial cells (*IECs*), lamina propria (*LP*) lymphocytes, and mesenteric lymph nodes (*MLN*). Levels were normalized to glyceraldehyde 3-phosphate dehydrogenase (*GAPDH*) (*n* = 5) (**P < 0.01). *EtOH* ethanol, *OXA* oxazolone

In order to look for the involvement of IL-25 in gut inflammation we used the oxazolone model of colitis, known by its clear type-2 immune phenotype [12, 37]. We used the recently described model of sensitization and challenge in order to ensure a pronounced colitis with minimal mortality in the mice [10]. We first analyzed IL-25 production in the gut following oxazolone instillation. Intestinal epithelial cells (IECs), LP lymphocytes, and the MLNs were isolated on day two post-challenge to look for IL-25 expression. IL-25 expression was significantly increased in IECs derived from colitic mice, and was also slightly elevated in the MLNs as compared with expression in the ethanol controls (Fig. 1). No difference in IL-25 expression was seen in the LP lymphocytes isolated from colitic mice in comparison to those derived from the ethanol controls (Fig. 1).

### Neutralizing antibodies to IL-25 and IL-17BR improve the clinical profile of colitis as well as the macroscopic appearance of colons

**Fig. 2:**
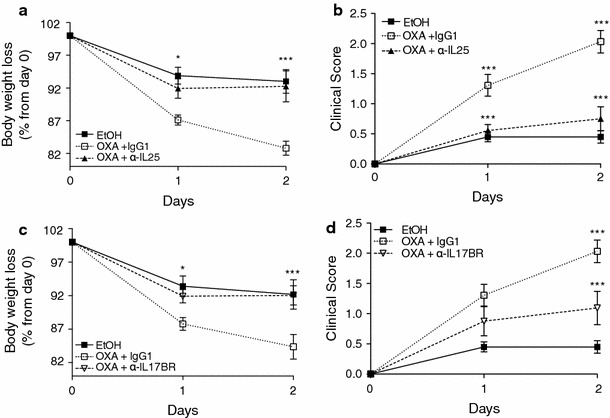
Neutralizing antibodies to IL-25 or IL-17BR ameliorate the onset of colitis. Mice were sensitized and challenged intrarectally with either oxazolone (OXA) or ethanol (EtOH). Anti-IL25 (**a**, **b**) or anti-IL17BR monoclonal antibodies (mAbs) (**c**, **d**) were given intraperitoneally before sensitization and challenge. The clinical onset of disease was evaluated by the loss of body weight (**a**, **c**) and the clinical disease index (**b**, **d**). Data from each antibody represent two separate experiments (each representative of two independent experiments) with 10–20 mice per group. The values represent means ± SEM (*P < 0.05/***P < 0.001)

**Fig. 3:**
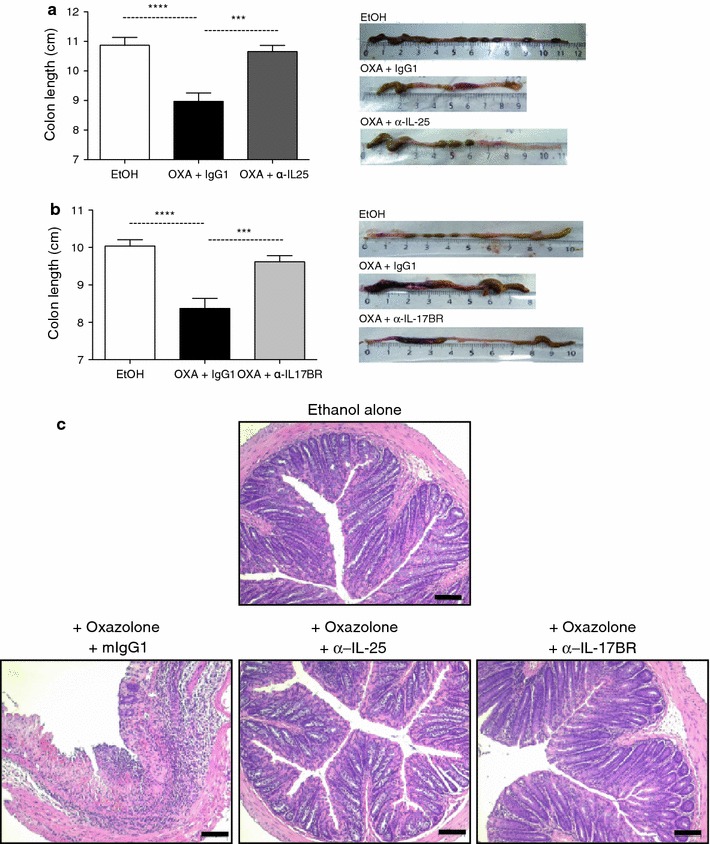
The macroscopic clinical signs of oxazolone colitis are reversed by treatment with neutralizing antibodies to IL-25 and IL-17BR. Analysis was performed on day 2 post-intrarectal injections, and the length of the colon and its macroscopic appearance were evaluated. Colon length and macroscopic appearance of colons from anti-IL-25- (**a**) and anti-IL-17BR-treated mice (**b**). Colon sections from each group were collected for H&E staining (×100) (**c**). *Black bars* represent 100 μm. Data from each antibody represent two separate experiments (each representative of two independent experiments) with 10 mice per group (****P < 0.0001)

In order to test the effects of blocking IL-25 signalling, administration of anti-IL-25 and anti-Il-17BR neutralizing antibodies was performed peritoneally 24 h prior to sensitization and challenge with OXA. Isotype control antibodies were administered as appropriate. Oxazolone challenged IgG1-isotype control treated mice exhibited a rapid onset of colitis, as early as 24 h post intrarectal injection, leading to a severe decrease in body weight and high clinical scores (Table 1). The colitic mice presented with bloody stools and had lost approximately 15 % of their weight by day 1 post-injection and around 18 % on day 2 (Fig. 2a, c). In contrast, weight loss was significantly reduced in the anti-IL-25- (Fig. 2a) and anti-IL-17BR-treated mice (Fig. 2c). Treated mice had also recovered most of their weight loss by day 2, in striking contrast to colitic animals (OXA+IgG1) that continued losing weight. Blocking IL-25 or IL-17BR also significantly improved the clinical phenotype of the mice, with treated mice presenting with reduced diarrhea and gross bleeding (Fig. 2b, d; Table 1). In addition, some of the colitic animals required euthanasia due to their disease symptoms reaching predetermined humane endpoints; this did not occur in anti-IL-25- and anti-IL-17BR-treated mice. Following these observations, we sought to look at the phenotypic changes in the intestine. One of the major macroscopic features of colitis in mice is the shortening of the colon that occurs due to ulceration and inflammation [38]. In order to assess the severity of colitis, we analyzed the macroscopic and microscopic appearance of the colons on day 2 post-challenge. We observed a shortening of the colon in the colitic mice (OXA+IgG1 isotype), which was almost completely reversed in the anti-IL-25- (Fig. 3a) or anti-IL-17BR (Fig. 3b) -treated mice. As expected, colons from colitic mice showed severe bowel edema and hemorrhage involving the distal 50 % of the colon (Fig. 3). Strikingly, in mice that received anti-IL-25 and anti-IL-17BR, compared with the untreated animals, the colons appeared to have less damage to the intestinal walls, less ulceration, and reduced bloody stools, similar to the findings in the ethanol controls. H&E staining of colon sections was performed to confirm our observations at a microscopic level. Colitic (OXA+IgG1) colons showed a disrupted epithelial cell layer due to extensive ulceration, accompanied by a reduction of the density of tubular glands and dense infiltration of the superficial layers of the mucosal tissue (Fig. 3c). However, anti-IL-25- or anti-IL-17BR-treated colons presented with very little inflammation (Fig. 3c). In summary, blocking IL-25 signalling via neutralizing antibodies against IL-25 or IL-17BR significantly improved the outcome of the disease and the macroscopic inflammation in the colon.

### Colitis-induced blood eosinophilia and serum IgE are less pronounced following IL-25 and IL-17BR neutralization

**Fig. 4:**
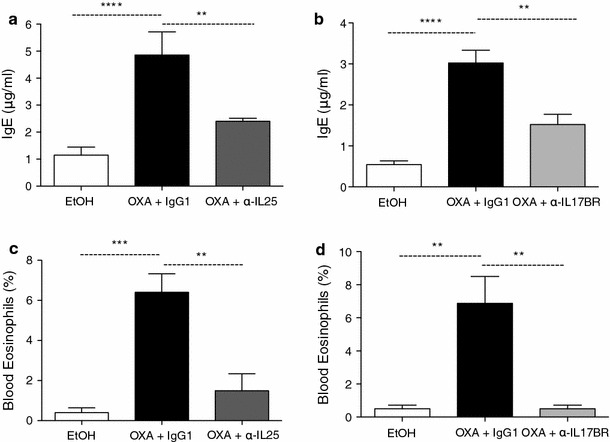
Eosinophilia and serum IgE levels are regulated by IL-25. IgE levels were measured by enzyme-linked immunoassay (ELISA) (**a**, **b**) and eosinophils were counted in slides of blood smears stained with Giemsa (**c**, **d**). Data are representative of two experiments (10 mice per group) (**P < 0.01)

Type-2 inflammatory responses are characterized by increased serum IgE and recruitment of eosinophils [39]. Administering IL-25 peritoneally has been shown to induce IgE isotype switching and eosinophilia [14]. For these reasons we sought to assess blood eosinophil levels in colitic mice as well as quantifying serum IgE. Blood and blood smears were collected on day 2 post-injection. Total IgE levels were quantified by ELISA and differential cell counts were performed on blood smears. Circulating IgE levels were significantly elevated in the OXA+IgG1 colitic mice in comparison with ethanol controls, but this increase was significantly reduced in mice administered with anti-IL-25 (Fig. 4a) or anti-IL-17BR (Fig. 4b). In contrast, the production of IgG2a, an isotype more typically seen in type-1 inflammation, remained unchanged in all the groups (data not shown). Additionally, anti-IL-25 and anti-IL-17BR treatment resulted in a significant decrease in the circulating numbers of eosinophils (Fig. 4e, f). Therefore, blocking IL-25 signalling improved some of the aspects of the inflammatory response, more specifically reducing plasma cell release of IgE and eosinophil mobilization.

### Blockade of IL-25 signalling down-regulates type-2 cytokines

**Fig. 5:**
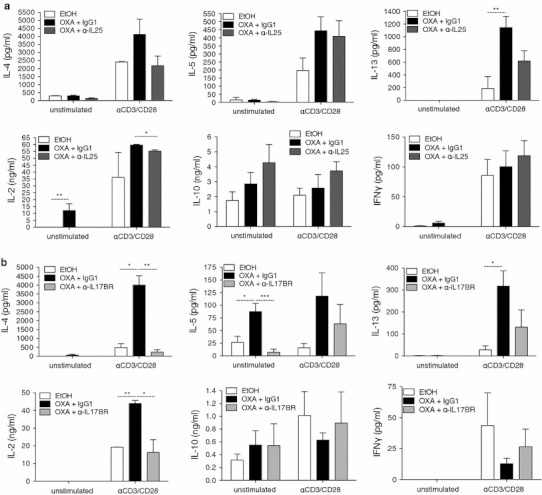
Cytokine production of in vitro unstimulated and anti-CD3/CD28-stimulated MLN cells. MLN cells were isolated on day 2 post-challenge and cultured for 48 h in the presence or absence of anti-CD3 and anti-CD28. Culture supernatants were analyzed by ELISA for IL-2, IL-4, IL-5, and IL-13 cytokines. Cytokine production by MLN cells from oxazolone mice treated with anti-IL-25 mAb (**a**) or anti-IL-17BR mAb (**b**). Data are representative of two independent experiments (*n* = 6). *IFNγ* interferon γ

**Fig. 6:**
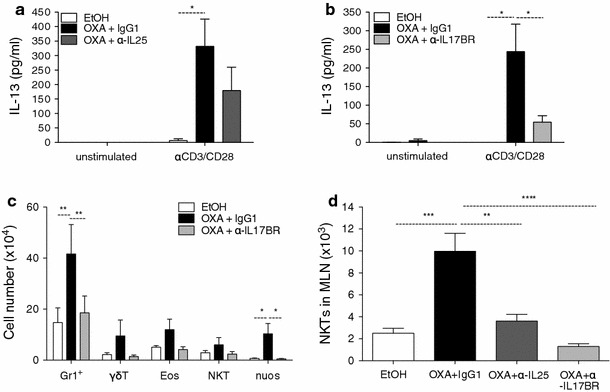
IL-13 production by lamina propria lymphocytes and innate cell infiltration are reduced in the absence of IL-25. Lamina propria lymphocytes were isolated on day 2 post-challenge and cultured for 48 h in the presence or absence of anti-CD3 and anti-CD28. Culture supernatants were analyzed by ELISA for the presence of IL-13 in LP cells from anti-IL-25-treated (**a**) or anti-IL-17BR-treated mice (**b**). Innate cell infiltration in the lamina propria, as analyzed by flow cytometry (**c**) and number of natural killer T (*NKT*) cells in the mesenteric lymph node (**d**). Data are representative of two independent experiments (*n* = 6).* Eos* eosinophils,* nuos* nuocytes

To investigate the cytokine profile in OXA colitic mice we isolated MLN cells on day 2 post-challenge and cultured single-cell suspensions for 48 h in the presence or absence of anti-CD3/anti-CD28. Cell supernatants were analyzed by ELISA to quantify cytokine levels. Anti-CD3/CD28 stimulated MLN cells from OXA+IgG1 showed increased levels of IL-4, IL-5, and IL-13 (Fig. 5a, b). Administration of anti-IL-25 or anti-IL-17BR reduced the type-2 cytokine production by MLN cells (Fig. 5). Consistent with the decrease in serum IgE and eosinophils, IL-4 production from anti-IL-25- and anti-IL-17BR-treated mice was substantially reduced (Fig. 5). IL-13 levels were also lower in the treated mice compared with the isotype control. This observation was also confirmed in the supernatants of lamina propria CD45^+^ cells (Fig. 6a, b), and more significantly so in anti-IL-17BR-treated mice (Fig. 6b). IL-2 production, associated with T-cell proliferation was, perhaps not surprisingly, increased in the colitic mice. There was a significant reduction in IL-2 production in cells derived from anti-cytokine antibody-treated mice. Despite this, no significant differences between the groups were seen in the numbers of CD4^+^ or CD8^+^ T cells in the MLN (data not shown). Some differences were noted in the basal level of cytokine expression that can be attributed to experimental variability. No differences in IFN-γ or IL-10 production were observed in any of the groups. Therefore, consistent with our previous observations, blocking IL-25 ameliorates inflammation in the gut by impairing harmful type-2 cytokine release. These observations are consistent with a role for IL-25 in regulating the expression of type-2 cytokines [14, 20], but are perhaps more significant for IL-13, as recent discoveries have shown that IL-25 is crucial for the induction of IL-13[25, 28].

### Impaired IL-25 signalling results in lower innate cell infiltration in the lamina propria and decreased numbers of NKT cells in the MLN

Following the observation of increased cell infiltration in H&E-stained colon sections, we looked at which cell types were responsible for the cytokine production and the driving of inflammation in the intestine. In the oxazolone colitic mice, the colon LP was flooded with inflammatory cell types, such as CD4^+^ T cells (data not shown), neutrophils, γδ T cells, eosinophils, NKT cells, and nuocytes (Fig. 6c). In keeping with our histology study, we observed almost no cell infiltration in the LP of mice treated with anti-IL-17BR (Figs. 3c, 6c). We also analyzed the different cell populations in the MLNs and found that NKT cells were the only cell type increased in the colitic mice in this tissue (Fig. 6d). However, this rise in NKT cell numbers did not occur in mice administered with anti-IL-25 or anti-IL-17BR. No difference between treated and colitic mice was seen in T-cell numbers or nuocyte numbers in the MLN (data not shown).

### IL-17BR^+^ nuocytes and NKT cells are the cellular sources of IL-13 in oxazolone colitis

**Fig. 7:**
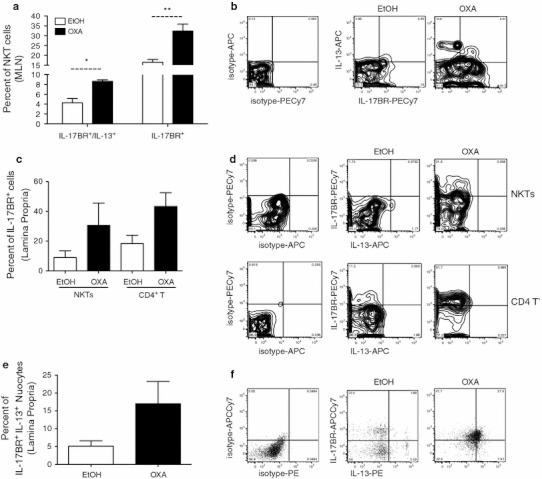
Nuocytes and NKT cells are the main cellular sources of IL-13 in oxazolone colitis. Percentages of IL-17BR^+^/IL-13^+^ and IL-17BR^+^ NKT cells in the MLN (**a**). Intracellular IL-13 cytokine production and surface expression of IL-17BR by NKT cells derived from the MLN of EtOH or OXA-challenged mice (**b**). Percentages of IL-17BR^+^ CD4^+^ T cells and NKT cells in the lamina propria (**c**); surface expression of IL-17BR in NKT or CD4^+^ T cells (**d**). Percentage of IL-17BR^+^ IL-13^+^ nuocytes in the lamina propria (**e**). Intracellular IL-13 cytokine production and surface expression of IL-17BR in the lineage-ICOS^+^ nuocyte population in EtOH- versus oxazolone-treated mice (**f**) (*n* = 10)

IL-13-producing NKT cells have previously been shown to drive inflammation in type-2 colitis, both in a mouse model and in human UC disease [10, 40]. Here we found that levels of IL-13 were increased in culture supernatants from MLN cells and LP lymphocytes from colitic mice. To determine the cellular sources of IL-13 during oxazolone colitis, we isolated LP and MLN cells on day two post-challenge and, using intracellular cytokine staining, examined which cell types expressed IL-13 by flow cytometry. In the MLN, the numbers of NKT cells increased following oxazolone instillation (Fig. 6d). In colitic mice, around 30 % of NKT cells expressed IL-17BR and 9–10 % of the IL-17BR^+^ NKT cells were also actively expressing IL-13 (Fig. 7a, b). Both IL-17BR^+^ and IL-17BR^+^ IL-13^+^ double-positive NKT cells were increased in the colitic mice compared with the EtOH controls (Fig. 7a). No IL-13 expression was detected in cell types such as CD4^+^ T cells and γδ T cells in the MLN, and although a proportion of CD4^+^ T cells was found to express IL-17BR, this population was not elevated compared to that in the ethanol controls (data not shown). In the LP, IL-17BR expression was found in around 40–50 % of CD4^+^ T cells and NKT cells from colitic mice compared to 20 % in EtOH controls (Fig. 7c). However, IL-13 production was not found in either of these cell types (Fig. 7d) or in γδ T cells (data not shown). In the mucosa, we found that the sole producer of IL-13 on day two following OXA instillation was the IL-17BR^+^ Lin^−^ ICOS^+^ nuocyte population (Fig. 7e, f). The proportion of IL-17BR/IL-13 double-positive nuocytes was also increased in the colitic mice compared with that in the ethanol controls (Fig. 7e). Thus, at the time point analyzed, NKT cells represented the major source of IL-13 in the MLN, while in the LP, this role was performed by nuocytes.

### Anti-IL-25 and anti-IL-17BR suppress IL-13 production by nuocytes in vitro

**Fig. 8:**
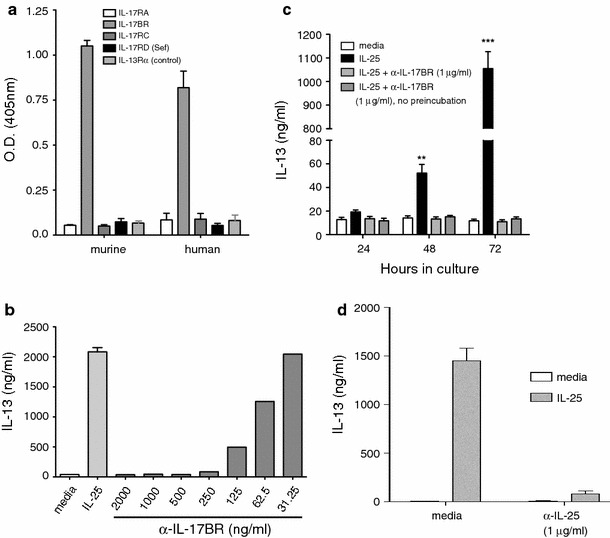
Anti-IL-17BR and anti-IL-25 inhibit the production of IL-13 by IL-25-stimulated nuocytes. Anti-IL-17BR antibody was screened for binding to IL-17 receptor family members and a control protein IL-13Rα-Fc (**a**). NBNT cells containing nuocytes were cultured in the presence of IL-25 and increasing concentrations of anti-IL-17BR, and IL-13 production was analyzed by ELISA at 72 h (**b**). NBNT cells were cultured for 24, 48, and 72 h in the presence of IL-25 and anti-IL-17BR with and without preincubation with the antibody, and analyzed for IL-13 production (**c**). IL-13 production by NBNT cells cultured for 72 h in the presence of IL-25 and anti-IL25 antibody (**d**). Data are representative of two independent experiments.* NBNT* nonBnonT cells, *O.D.* optical density, *Sef* similar expression to FGF

Nuocytes have been described recently as the main innate IL-13-producing cell type that is critical for the development of type-2 immune responses in helminth infection and allergic lung inflammation [28, 41]. In this study we observed an increase in nuocyte infiltration in the LP of colitic mice that was inhibited by treatment with anti-IL-17BR. We also found that nuocytes were the main IL-13-producing cell type in the LP. Therefore, we sought to investigate the effect of blocking IL-25 signalling in in vitro cultures of nuocytes. Nuocytes were cultured in the presence or absence of IL-25. Cell cultures were also incubated with anti-IL-17BR or anti-IL-25 for 24–72 h. We found that anti-IL-17BR antibody inhibited IL-13 production by nuocytes in a dose-dependent fashion (Fig. 8b) and that this inhibition occurred regardless of whether the cells were preincubated with the anti-IL-17BR antibody prior to the addition of IL-25, or whether the antibody was added at the same time as IL-25 (Fig. 8c). To discard possible unspecific binding of the antibody we screened against other IL-17 receptor family members. We found that anti-IL-17BR was highly specific for the mouse and human IL-17BR receptors, but showed no cross-reactivity with other IL-17 receptor family members (Fig. 8a). Anti-IL-25 antibody has been previously shown to have a therapeutic effect in lung allergy models [23, 25]. Here we show that, similar to the anti-IL-17BR antibody, anti-IL-25 is also capable of inhibiting IL-13 production from nuocytes in vitro (Fig. 8d).

## Discussion

We demonstrate a novel role for IL-25 as a pro-inflammatory cytokine in a type-2 model of colitis. Neutralizing IL-25 prior to the onset of colitis almost completely reversed the physical symptoms of disease. Administration of anti-IL-25 or anti-IL-17BR prevented the severe weight loss typical of colitic mice and significantly improved their clinical phenotype. This protective effect was shown to be even more striking upon close analysis of the colons. The oxazolone model of colitis typically affects the distal half of the colon [32] and leads to profound changes in the intestine, including colon shortening due to ulceration [38] and innate cell and lymphocyte infiltration to the superficial layer of the mucosa [11]. We found that neutralizing anti-IL-25 or anti-IL-17BR antibodies protected against this colon shortening, intestinal bleeding, and disruption of the mucosal structure. The histology sections showed perhaps the most obvious difference, with colons from treated mice presenting with a normal appearance (no changes of the mucosal wall or cell infiltration).

At a cellular level, we observed intense immune cell infiltration of the lamina propria (LP) in the colitic mice, with cells such as neutrophils, eosinophils, and T cells, but also NKT cells, γδ T cells, and, most notably, nuocytes. This general cell infiltration was not observed when we prevented IL-25 signalling by blocking the IL-17BR receptor. Moreover, we found a similar trend for the increase in NKT cells, but not for other cell types, in the MLN. This increase of the NKT population in both the LP and MLN has a particular interest, as it correlates with previous reports in UC patients [40]. Also in oxazolone colitis, NKT cells have been proposed as the effector cell type driving type-2-like inflammation. In those studies, CD1d-deficient mice and invariant NKT (iNKT)-deficient mice that lacked NKT cells were shown to be protected from the development of gut inflammation [10]. Studies in lung allergy also showed that IL-25 could activate a population of IL-17BR^+^ NKT cells which resulted in Th2 cell activation and eosinophilia [18]. In line with these previous findings, our data suggest that IL-25 plays a similar role in the gut by activating IL-17BR^+^ NKT cells to produce IL-13 and that blocking IL-25 signalling has a profound positive impact in resolving the intestinal inflammation.

In the case of γδ T cells, their specific role in colitis has not yet been defined. Recent reports have found contradictory data on the protective or exacerbating role of γδ T cells in the intestinal mucosa, depending on which colitis model was studied [42]. TCR-α chain-deficient mice develop a type-2 colitis that is driven by γδ T cells [43], but both γδ T cells and αβ T cells were found to have accumulated in the inflamed regions of CD and UC patients [44]. We found here that blocking IL-25 signalling using anti-IL-17BR decreased the numbers of γδ T cells in the LP, but whether this was due to a direct effect on their proliferation or to the decreased general level of inflammatory cytokines cannot currently be defined. Most interestingly, we also found that the number of lineage-negative, ICOS^+^ cells (or nuocytes) in the LP was increased in the colitic mice, as compared to findings in mice treated with the anti-IL-17BR neutralizing antibody and the ethanol (EtOH) controls. In addition, we found that, in the LP, IL-17BR^+^ nuocytes were the sole cellular source of IL-13 at the time point where inflammation was assessed and that both anti-IL-25 and anti-IL-17BR blocking antibodies inhibited IL-13 production by nuocytes in vitro. Nuocytes have been described recently as a new innate cell type that is responsive to IL-25 and is the main producer of IL-13 in the gut during helminth parasite infection [28] and here we found for the first time the presence of this new type-2 innate lymphoid cell (ILC) during inflammation in colitis. Thus, in addition to the NKT cell IL-13 production, a mechanism of action for IL-25 during type-2 colitic inflammation can now start to be defined. Intestinal epithelial cells (IECs) are induced to secrete IL-25 by disruption of the gut microenvironment following oxazolone instillation. IL-25 then activates IL-17BR^+^ nuocytes in the LP to produce IL-13 and these events initiate the type-2 inflammatory cascade. It is not surprising to find that IECs are the main producers of IL-25 in the gut because these cells form the first barrier of protection against gut pathogens. Moreover, this observation is consistent with other studies, where IL-25 expression was up-regulated in small intestine epithelial cells, rather than in LP immune cells following nematode infection [45].

Consistent with a role for IL-25 upstream of type-2 cytokines [10, 11, 37], we found that blocking IL-25 or IL-17BR had a profound effect on the type-2 cytokine profile, and that IL-4, IL-5, and IL-13 production was reduced in the antibody-treated mice. IL-13, IL-4, and IL-5 blocking strategies have been found to ameliorate oxazolone colitis [10, 11, 46] and in this study we found that neutralizing IL-25 produced a similar protective effect. We believe that IL-25 acts upstream of these type-2 cytokines as an initiating force of the immune response. In the human disease, IL-13 has been defined as an important cytokine driving the pathogenesis of UC [40], being expressed by cytotoxic NKT cells and disrupting the epithelial layer of the colon, which explains the colonic phenotype of UC [6]. In addition, more recent studies have again underlined the type-2 phenotype of UC, showing that yet another type-2 initiator cytokine, IL-33, is involved in the development of inflammation in the gut [33, 34, 47]. IL-33 has also been described to play a role in *T. muris* infection and has been shown to induce a type-2 cytokine profile in the intestine [48]. Although we did not find IL-33 expression in this particular study (data not shown), the fact that IL-25 and IL-33 lead to similar inflammatory outcomes in the intestine may mean that they are good targets for potential therapy in UC patients, upstream of the type-2 cytokine cascade.

IL-25 can have both anti- and pro-inflammatory roles in the gut, depending on the type of inflammation that is present. In type-1 responses, such as in DSS and TNBS colitis, IL-25 clearly plays an anti-inflammatory role [16, 29]. In previous studies, IL-25 was shown to have a protective effect towards the development of colitis by down-regulating IL-12 and IL-23, which, in turn, limits the Th1-driven inflammatory response. More recently, this IL-25 protective role has been associated with the activation of alternatively activated macrophages with anti-inflammatory properties [30]. Consistent with these findings, chronically infected IL-25-deficient mice develop severe intestinal inflammation that is Th17-driven [31]. However, none of these studies looked in-depth into specific type-2-driven intestinal responses, such as the one we used here. Indeed, in type-2-skewed responses, such as some parasite infections in the gut, IL-25 clearly drives the inflammation upstream of IL-4, IL-5, and IL-13 and therefore acts as a pro-inflammatory cytokine [27, 28, 31]. These studies are consistent with our model, where blocking IL-25 signalling using neutralizing antibodies ameliorated the type-2 inflammation in the gut. Our data add a new key player in type-2 colitis, showing that IL-25 can act as a potent pro-inflammatory cytokine in this model.
